# Supplementary figures and images for: Collective Emotions Online and Their Influence on Community Life
Source: PLoS One. 2011 Jul 27;6(7):e22207. doi: 10.1371/journal.pone.0022207 (PMC3144870; doi:10.1371/journal.pone.0022207)

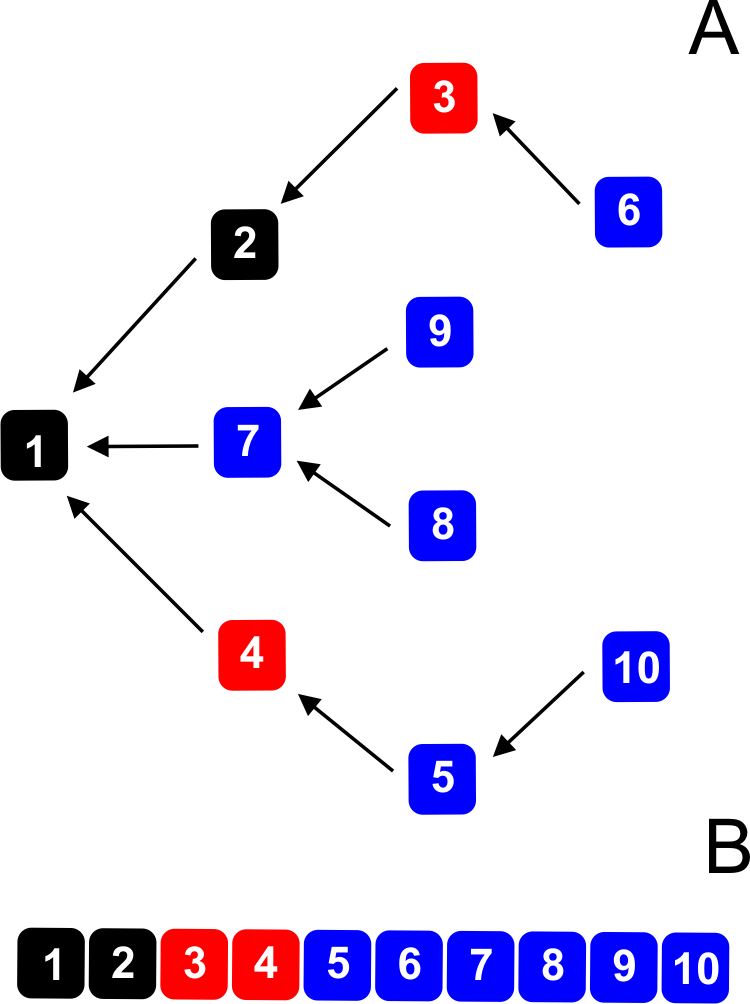

Supplement: Figure S1 — The difference between the actual tree structure (A) present in the BBC and Digg datasets as compared to the chronological layout of the posts (B). The numbers indicate the order of messages (1 being the first, 10 being the last) while arrows indicate that a post was given in reply to another one (e.g. post 9 is the response to post 7). (TIF) [file pone.0022207.s001.tif]

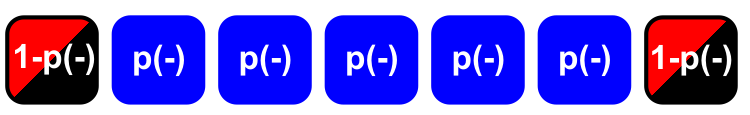

Supplement: Figure S2 — In case of the i.i.d. random process to obtain the probability of finding a cluster of exactly consecutive emotional values (here and ) one has to take into account two factors: the length of the cluster itself and the issue that on the both borders there should be posts with emotional value other than inside the cluster. Thus in the presented case the probability is proportional to . (TIF) [file pone.0022207.s002.tif]
