# Supplementary material for: Collective Emotions Online and Their Influence on Community Life
Source: PLoS One. 2011 Jul 27;6(7):e22207. doi: 10.1371/journal.pone.0022207 (PMC3144870; doi:10.1371/journal.pone.0022207)
Supplement: Text S1 — includes a detailed derivation of the analytical approximation of cluster length distribution as well as comparison of this approach with the i.i.d. random process, Markov chain and Markov model. It also contains the analysis of the influence of the thread length on the emotional cluster distributions and discussion on the number of unique users in emotional clusters. (PDF) [file pone.0022207.s003.pdf]

Collective Emotions Online and Their Influence on Community Life – Supporting Information File S1

Anna Chmiel, Julian Sienkiewicz, Mike Thelwall, Georgios Paltoglou, Kevan Buckley, Arvid Kappas and Janusz A. Hołyst

S1 Data structure. The three datasets on which sentiment analysis was performed are characterized by different structures. In the case of Blogs06, all posts were originally arranged in chains of successive comments, as shown in Fig. S1B. In other words new posts are automatically added after the last post. On the other hand, the BBC and Digg data are arranged in a forum-like structure (see Fig. S1A), meaning that each user may make a comment to any previous post, thus starting a separate discussion. However, for the purpose of this study each discussion (thread) in each dataset was arranged chronologically, as in Fig. S1B. In this way it was possible to compare these different communities. Although BBC and Digg have a forum-like structure, the default view presented to the user was chronological. Thus a chronological simplification for analysis can be justified.

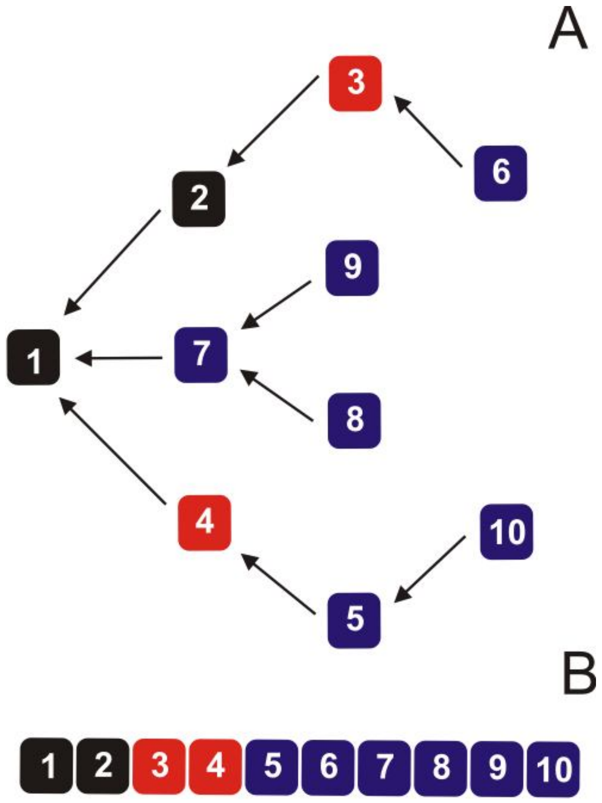

Fig. S1. The difference between the actual tree structure (A) present in the BBC and Digg datasets as compared to the chronological layout of the posts (B). The numbers indicate the order of messages (1 being the first, 10 being the last) while arrows indicate that a post was given in reply to another one (e.g. post 9 is the response to post 7).

S2 Emotional cluster distributions. An independent and identically distributed (i.i.d.) random process [1] corresponds to the simplest stochastic process where there is no statistical dependence between events at consecutive time-steps and at every time-step the event probability distribution is the same. When the parameter *p(e)* describes the probability of negative, positive or neutral emotion then the probability to find a cluster of size *n* among all clusters of the same valence *e* (see Fig. 2) scales as $[1-p(e)]^2 p(e)^n$. Here the factor *[1-p(e)][1-p(e)]* corresponds to an event other than *e* just before and just after the cluster, i.e., where a change in valence takes place (for details see Fig. S3). Taking into account the normalization $\sum_{n=1}^{n=\infty} P_{i.i.d.}^{(e)}(n) = 1$ condition we have

$$A\sum_{n=1}^{n=\infty}[1-p(e)]^2 p(e)^n = A[1-p(e)]p(e) = 1 \qquad \text{(S1)},$$

where *A* is the normalization constant (the sum starts from *n=1* as we do not take into account clusters of size 0). Using simple algebra we obtain the cluster distribution $P_{i.i.d.}^{(e)}(n) = [1-p(e)]p(e)^{n-1}$ and the resulting cumulative distribution is

$$P_{i.i.d.}^{(e)}(\geq n) = p(e)^{n-1} \qquad \text{(S2)},$$

as represented by a dotted line in Fig. S2.

A Markov chain [2] is a basic stochastic process with one memory step when the probability of the next time state depends only on the previous one by corresponding conditional probabilities. The probability of finding a cluster of size *n* scales as $[1-p(e)][1-p(e|e)]p(e)p(e|e)^{n-1}$ and, similarly to the i.i.d. process, the factor *1-p(e)* corresponds to any event just before the cluster other than *e* and the factor *1-p(e|e)* to the event just after the cluster. Taking into account the normalization condition (following the same scheme as in the i.i.d. case) we have the cluster distribution $P_{M.}^{(e)}(n) = [1-p(e|e)]p(e|e)^{n-1}$. Finally the cumulative distribution is

$$P_{M}^{(e)}(\geq n) = p(e\,|\,e)^{n-1} \qquad \text{(S3)},$$

as represented by a dashed line in Fig. S2.

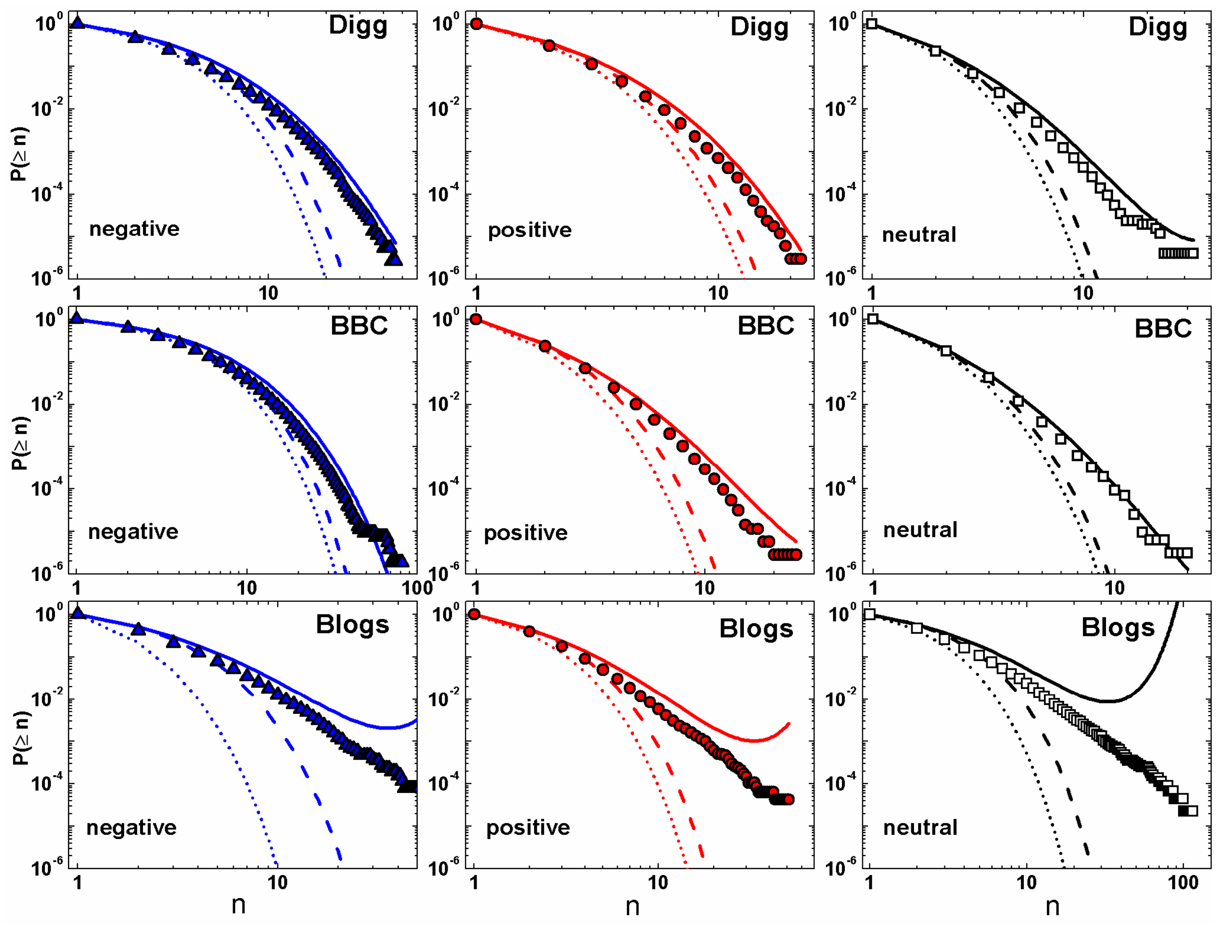

Fig. S2. Cumulative distribution of the cluster size for all data used in the study. Symbols are data (blue triangles, red circles and white squares, respectively for negative, positive and neutral clusters), dotted lines are i.i.d. processes given by Eq. (S2), dashed lines are Markov processes given by Eq. (S3) while solid lines come from Eq. (S6) and represent distributions based on the preferential attraction rule. The spurious increase of $P^{(e)}_{\alpha}$ *(≥n)* for $n \geq 40$ for Blogs data is due to violation of the scaling (S4).

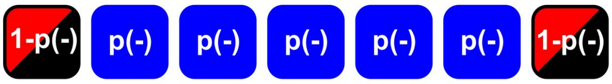

Fig. S3. In case of the i.i.d. random process, to obtain the probability of finding a cluster of exactly n consecutive emotional values (here $n=5$ and $e=-1$) one has to take into account two factors: the length of the cluster itself and the issue that on the both borders there should be posts with emotional values other than inside the cluster. Thus, in the presented case, the probability is proportional to $[1-p(-)]p(-)^5[1-p(-)]$.

The conditional probability $p(e|ne)$ that expresses the chance that after n messages with the same emotional valence e the next post will continue this trend is shown in Fig. S4 as symbols. The phenomenon of preferential attraction

$$p(e \mid ne) = p(e \mid e) n^{\alpha} \tag{S4}$$

is evident for all datasets. The range of this scaling varies for different e-communities and different emotional valences of clusters – e.g., for the BBC neutral clusters we find a good fit for the whole range of the data. Note that the scaling can not be valid for very large n since *p(e|ne)* must be smaller than 1. Preferential attraction plays a crucial role for cluster distribution. An approximation to real data behavior can be obtained by extending the relation (S4) up to the maximal cluster size in the considered community. The resulting cluster probability distribution for clusters of the size n scales as $[1-p(e|e)n^{\alpha}][p(e|e)]^{n-1}[(n-1)!]^{\alpha}$ where the first factor corresponds to an event other than *e* just after the cluster of size *n*. This result resembles the previous Markov formula with additional factors reflecting the preferential effect. The analytical form of the normalization factor can be obtained only as an approximation when

$$p(e\,|\,e)^{n_{\max}}\left(n_{\max}!\right)^{\alpha}\approx 0 \qquad \text{(S5)}$$

The relation (S5) is fulfilled provided that n_{max} is not too large ($n_{max}<40$) or α is small ($\alpha<0.1$). These conditions hold for all of the data sets except for the neutral clusters in Blogs06. As a result, the cumulative cluster distribution is

$$P_{\alpha}^{(e)}(\geq n)\approx p(e\,|\,e)^{n-1}\left[(n-1)!\right]^{\alpha} \qquad \text{(S6)}$$

This approximation is presented in Fig. S2 with solid lines. The fit to the data is far better than in the case of the i.i.d. assumption or even than the Markov approach, especially for large *n*. The observed differences between (S6) and the real data come from the artificial extension of the scaling relation (S4). It leads to the spurious behavior of $P^{(e)}_{\alpha}(\geq n)$ when it increases for large n (see Blogs06 clusters in Fig. S2).

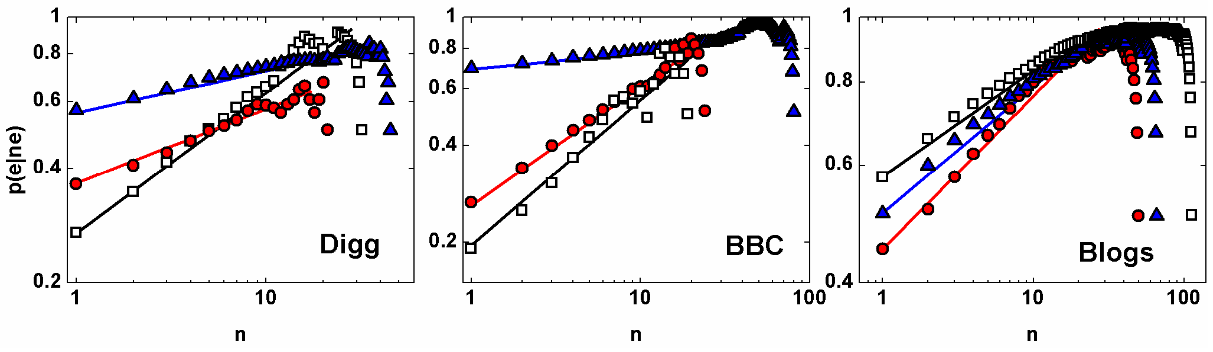

Fig. S4. The conditional probability *p(e|ne)* of the next comment occurring having the same emotion for Digg, BBC and Blogs06 data. Symbols are data (blue triangles, red circles and white squares, respectively for negative, positive and neutral clusters) and lines reflect the fit to the preferential attraction relation (S4).

S3 Conditional Probability. In Fig. S5 we investigate more carefully the power-law character of the conditional probability *p(e|ne)*. We are aware of the fact that the regime of power law scaling is very short, although a comparison between power-law and linear fits, shows that the first function is in much better agreement with the data. Especially for the first point we can observe a large divergence between those fits, which is significant taking into account the fact that the first point has the best statistical quality (i.e., the number of events at $n=1$ is much larger than for $n>10$).

	Digg			BBC			Blogs		
e:	+	0	-	+	0	-	+	0	-
p(+|e)	0.37	0.30	0.27	0.27	0.19	0.14	0.45	0.32	0.27
p(0|e)	0.20	0.27	0.17	0.15	0.20	0.17	0.39	0.58	0.22
p(-|e)	0.43	0.42	0.56	0.58	0.61	0.69	0.16	0.10	0.51

Table S1. The conditional probabilities for all datasets Each cell gives a conditional probability $p(e_2|e_1)$ of two post with emotions e_1 and e_2. For example for Digg data $p(+|0)=0.30$.

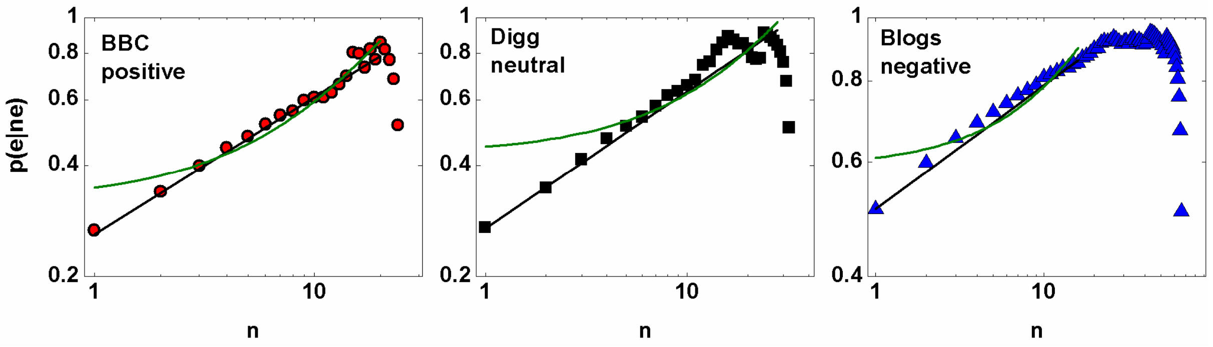

Fig. S5. A comparison between the power-law and linear fits to the conditional probability *p(e|ne)* for Digg neutral, BBC positive and Blogs06 negative posts. Symbols are data (blue triangles, red circles and black squares, respectively for negative, positive and neutral clusters), black lines reflect the fit to the preferential attraction relation (S4) and green lines are linear fits in the same regime.

S4 Influence of the thread length on the emotional cluster distributions. In all three cases the discussion lengths vary considerably. Figure S6 presents thread length distributions for BBC (left), Blogs (center) and Digg (right) data. The majority of discussions are shorter than 10 comments (this in not the case for Blogs where there are only threads with at least 100 comments – see the *Data sets* paragraph in the *Materials and Methods* section in the main text), yet there is also a significant number of very long threads in the fat-tail part of all three distributions. However, it is essential to notice that the thread length L is not directly connected to the maximal cluster size observed in the data. Figure S7 presents a comparison between the BBC cumulative cluster distribution calculated for all data and subsets characterized with different thread lengths ($L=20$, $L=50$ and $L=100$). As one can see, for the negative posts, the maximal cluster size in threads with $L=50$ is even longer than in the case of $L=100$.

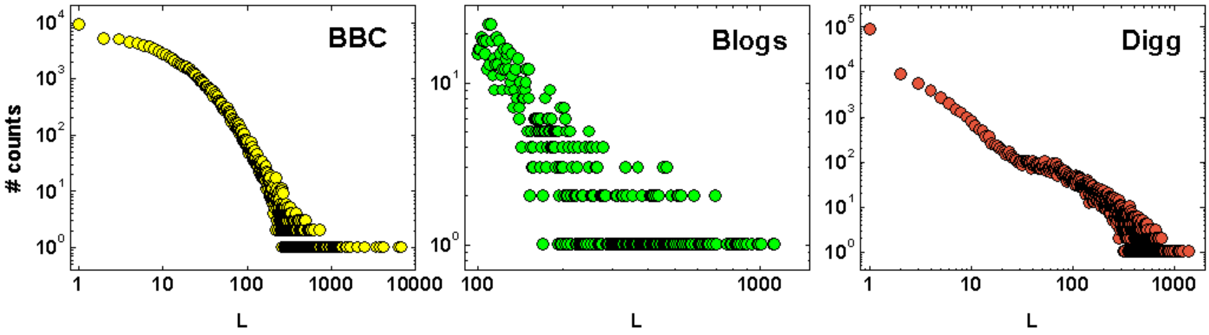

Fig. S6. Thread length distributions of the BBC (left) [3], Blogs (center) and Digg (right) data.

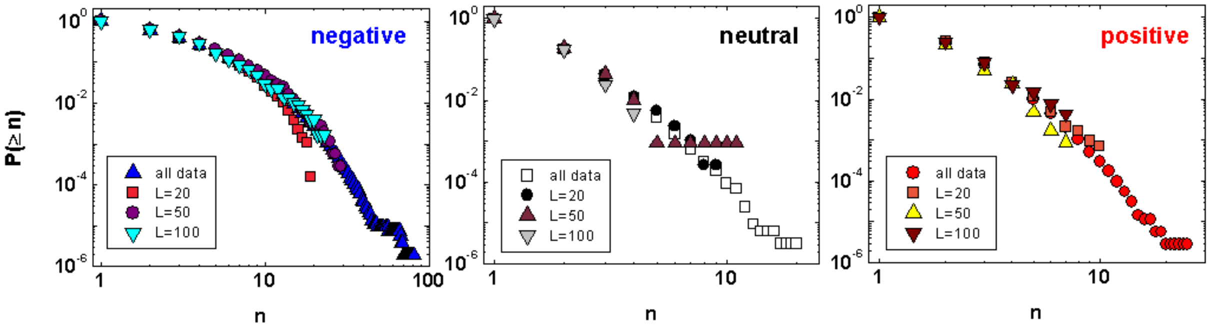

Fig. S7. Cumulative cluster distribution of the BBC negative (left), neutral (center) and positive (right) data for different subsets of thread lengths L.

S5 Unique users in clusters. To prove the validity of the main aspect of the paper – the transmission of emotions between discussion participants – it is crucial to check if the long threads are not dominated by a single user or just two people. Figure S8 shows the average number of unique users $\langle U\rangle_n$ (i.e., users with a unique id) that take part in emotional clusters of size n. It suggests that although the longer discussions are dominated by a limited number

of unique users, the majority of clusters is still created by a significant number of unique users (e.g., for negative clusters of size n=10 there are an average of 6 unique users).

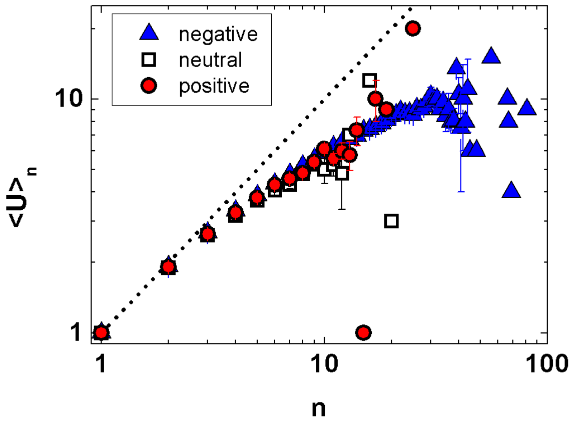

Fig. S8. Average number of unique users in the cluster $<U>_n$ versus the size of the cluster for BBC negative (triangles), neutral (squares) and positive (circles) data. The dotted line marks the relation $<U>_n=n$.

S6 Comparing the cluster distribution with the k-1 Markov model. Brendel at al. [4] used a *k-1* Markov model where the occurrences of a specific *k*-element sequence of nucleotides were obtained with information from the *k-1* previous events. The difference between the expected number of sequences and the observed value shows that, in the case of nucleotides, a Markov chain of order less than 4 does not give a good agreement.

To test how a *k-1* Markov model can be applied to the emotional statistics we defined the normalized difference *std(n)* between the expected and observed number of sequences with uniform emotional valence (an n -sequence is a chain of n messages with the same emotional valence; it is not the same as an n-cluster because, for example, sequences of size 4 can be part of a cluster of size 10):

$$std(n) = \frac{(Ns(n) - Es(n))}{\max\{\sqrt{Es(n)},1\}} \qquad \text{(S7)}$$

Where the *Ns(n)* is the number of observed sequences of size n, and *Es(n)* is the expected number of size n obtained according the equation:

$$Es(n) = Ns(n-1)p(e \mid n-2)\,. \qquad \text{(S8)}$$

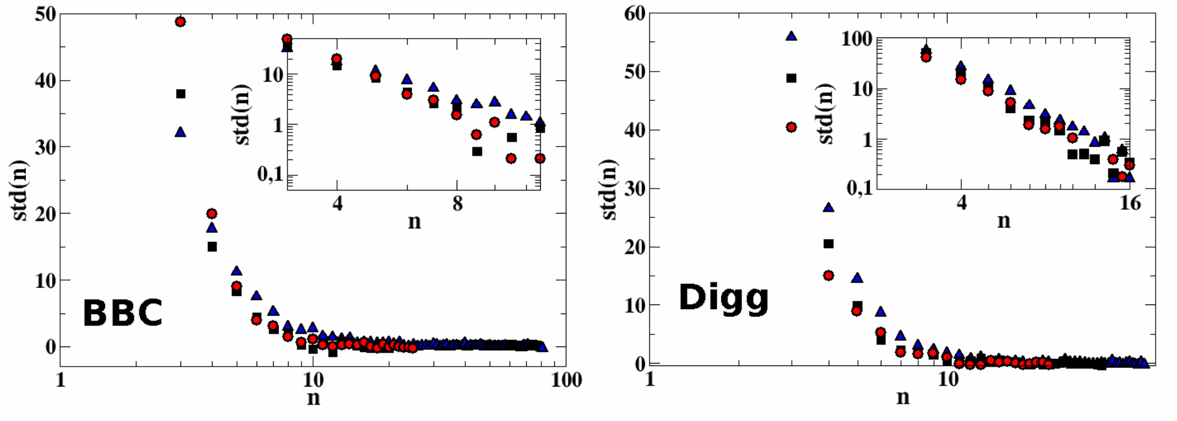

Fig. S9 The normalized difference *std(n)* (S7) between the observed number of sequences of size *n* and the number expected by the *k-1* Markov model versus the size of the sequence *n* for BBC(left) and Digg (right). Blue triangles show negative sequences, red circles represent positive sequence and the black squares are neutral sequences. The insets shown in a log-log scale focus on the first part of relation where *std(n) >0* and the power law relation between *std(n)* and *n* can be observed. Only sequences larger than 3 can be handled with this method [5].

The results, presented in Fig S9, allow us to conclude that in the case of emotional sequences a Markov model of order less than 10 does not perfectly fit the data. This is another argument proving that a long rage correlation exists in building the sequences. Moreover, it should be underlined that the *k-1* Markov model uses almost full information from the data, contrary to the method presented in our paper.

References

[1] Feller W (1968) *An Introduction To Probability: Theory And Its Applications* (Wiley, New Jersey)

[2] Norris JR (1997) *Markov Chains* (Cambridge University Press, Cambridge)

[3] Chmiel A, Sobkowicz P, Sienkiewicz J, Paltoglou G, Buckley K, Thelwall M, Holyst JA (2011) Negative emotions boost users activity at BBC Forum, *Physica A* 390, 2936.

[4] Brendel V, Beckmann JS, Trifonov EN (1986) Linguistics of Nucleotide Sequences: Morphology and Comparison of Vocabularies, *J Biomol Struct Dyn* 4: 011.

[5] Sinatra R, Condorelli D, Latora V (2010) Networks of Motifs from Sequences of Symbols *Phys Rev Lett* 105: 178702
